# Taking Severe Acute Malnutrition Treatment Back to the Community: Practical Experiences from Nutrition Coverage Surveys

**DOI:** 10.3389/fpubh.2016.00198

**Published:** 2016-09-13

**Authors:** Lenka Blanárová, Eleanor Rogers, Carine Magen, Sophie Woodhead

**Affiliations:** ^1^Action Against Hunger UK, London, UK

**Keywords:** community, community-based management of acute malnutrition, severe acute malnutrition, coverage, participatory

## Abstract

The community-based management of acute malnutrition treatment model was introduced to respond to the limited coverage of the inpatient model. Yet until the introduction of quick and low-cost approaches to measuring coverage, its reach was unknown. Once the Coverage Monitoring Network (CMN) had been created to roll out the routine measurement of direct coverage estimates to implementers, they found that programs were reaching only a third of cases. The barriers found to be limiting coverage were the result of the limited perceived value, and therefore focus, on the community. Therefore, the Network used the coverage assessment methodology as a way to encourage implementers to engage more fully with the community. By introducing small changes to the project cycle, specifically a participatory approach to assessments, program design and implementation, the CMN has changed the way implementers engage with the community. Instead of viewing them as passive receivers of services, they have shifted their perspective to view them as service delivery partners. The process provides implementers with a deeper understanding of the context while allowing the community to better understand the program, its challenges, and the identification of solutions. The Network observed implementers from Ministries of Health, and non-governmental organizations, adjusted their understanding and approach to service provision, which is critical if we are to see sustainable increases in program coverage. These experiences show that there is an appetite from implementers in multiple contexts for these practical and simple tools for re-engaging the community.

## Introduction

The community-based management of acute malnutrition (CMAM) treatment model, formerly known as community-based therapeutic care (CTC), was first introduced in 2000 in response to the limited effectiveness of the existing inpatient model (1). Programs require good clinical outcomes (high cure rates and low death rates) combined with high coverage rates to ensure high impact. Hospital-based treatment, although performing well clinically, had low coverage rates, resulting in only 10% of those children suffering from severe acute malnutrition (SAM) receiving treatment, when performing at their best (2). The low coverage rates were largely due to the high opportunity cost associated with receiving inpatient treatment, long distances to access care, and the risk of cross-infection, leaving many families refusing care (1, 3, 4).

The CTC model, later termed CMAM when endorsed by the World Health Organization and the United Nations in 2007, was designed to overcome many of these barriers while continuing to perform well clinically (5). There are four components to the model – inpatient and outpatient treatment for SAM, outpatient treatment for moderate acute malnutrition (MAM), and community mobilization. Since 2007, the approach has been adopted by ministries of health in over 70 countries, currently treating around 3 million children a year worldwide (6). Early programs implemented during the research phase recorded coverage rates of 70–80% (7) for the SAM outpatient component, which combined with good clinical outcomes resulted in high impact interventions. However, resource-intensive approaches to measuring coverage (centric systematic area sampling) meant that coverage was not routinely monitored. Therefore, although anecdotally it was understood that outpatient programs failed to continue reaching these initial high coverage rates, there was little evidence to back up this belief.

## Coverage Monitoring Network

In 2010, the semi-quantitative evaluation of access and coverage (SQUEAC) and the simplified lot quality assurance sampling evaluation of access and coverage (SLEAC) (8) approaches were created to address the limitations mentioned above. These quick and low-cost approaches directly measure program’s treatment coverage, allowing for routine measurements by program implementers. With these tools available, the Coverage Monitoring Network (CMN) was created (9). This inter-agency initiative, funded by European Commission and United States Agency for International Development between 2012 and 2016, was initially created with the sole aim of introducing the routine measurement of direct coverage estimates into community-based SAM program implementation.

The first phase of the project (2012–2014) trained program implementers in how to conduct a SQUEAC/SLEAC, identifying barriers and boosters to access and a coverage rate. From this program-level data, a global database was created providing data on the most frequently reported barriers to access and the median coverage rates. Programs were found to be reaching only a third of cases (10). The two most common barriers to access for services, across Africa, Asia, and the Americas, are a lack of awareness of malnutrition and of the program itself, both of which reflect limited community engagement (10). High opportunity costs,^1^ inter-program interface problems,^2^ and previous rejection from the program^3^ make up the five most frequently reported barriers (10). The majority of the barriers to access has been identified as the result of the limited perceived value and thus focuses on the “community” component of the implementation model. For that reason, a refocus on this element was the next logical way to sustainably improve coverage, returning to using the CMAM model as it was originally intended.

Through supporting program implementers in the assessment and the development of recommendations for program improvement, the CMN recognized the need for closer and more consistent support of implementers in the domain of community engagement. Hence, they worked with implementers across nine priority countries in Africa and Asia^4^ to re-orientate their understanding of the model and of the important role played by the community, viewing them not as beneficiaries but as partners. By engaging communities at the assessment stage of the program cycle, *via* the SQUEAC method, it opened the door for a continued dialog, involving key community stakeholders in successive discussions about the assessment findings, development of solutions, and crucially implementation of these solutions.

Based on experience from over 50 SQUEAC assessments, the CMN has found that communities need to participate in all stages of the project cycle, accompanying program implementers not only in the learning but also decision-making process. As a result, communities gradually assume their rightful role as partners in the service delivery, also moving toward a rights-based approach to programing, where the community is more empowered to hold the service providers to account. The community is subsequently actively contributing to the success of the program, which would otherwise be difficult to achieve without their intervention.

## Refocus on the Community – Learn, Analyze, and Act

### Learn: Participatory Research

Understanding the community is the first, essential step in community engagement. It should not be limited to understanding how the community perceives and interacts with the program but should also cover community structures, communication channels, and decision-makers. This took place during the qualitative stage of SQUEAC assessments^5^ through interviews and focus group discussions with a range of community figures, such as carers of malnourished children, traditional healers, community leaders, community-based organizations, or any other community members, reflecting the local diversity of ethnicity, gender, and religion. By engaging with multiple stakeholders, the aim is to ensure that all perspectives are captured, assenting or dissenting, with the aim of service delivery improvement clearly stated so that participants feel able to express negative opinions. In addition, the SQUEAC method is an iterative process, with each iteration being a direct result of the community dialog and exchange, during the qualitative phase of the assessment. Therefore, the process of participant selection is designed to capture a broad range of community members. It is also encouraged to acquire additional respondents in order to enable further exploration of a certain topic raised by the initial participants.

#### Analysis of Community Structures

The analysis of social structures identified various community actors, their areas of influence, and the allocation of decision-making powers within the family and/or the community. The understanding of the role of each member allowed programs to approach people or categories of people, who were in the best position to assume specific roles in future community engagement strategies. This also applied to the so-called gatekeepers, often opinion leaders, who could at any time, depending on their own perception of the program, “open or close the door” in terms of people’s understanding or access to the program. In many Muslim countries, including Burkina Faso (11), Chad (12), and Mali (13), religious authorities such as imams were often identified as these gatekeepers. Therefore, programs initiated or strengthened their interactions with them in order to harness their influence in the community. In Chad, for example, sensitization activities targeting imams resulted in further awareness raising about malnutrition through these highly respected figures – and, as a result, also reduced the volume of treatment refusals by fathers of malnourished children.

#### Analysis of Communication Channels

The understanding of existing community structures goes hand in hand with the knowledge of available communication channels as the insufficient awareness of malnutrition and/or CMAM program modalities can be easily traced to inadequate communication strategies developed and initiated by local health systems or supporting non-governmental organizations (NGOs). The majority of coverage assessments demonstrated that communication strategies focused on a one-way diffusion of relevant key messages. Communities were rarely engaged in two-way exchanges nor were important figures asked to endorse the messaging or share their experience of “struggle and success.”^6^ Although “traditional” awareness raising may be appropriate in the early stages of the program, it needs to be tweaked into a more participatory approach soon after so that the community starts using existing influential figures and channels available to them for the program’s benefit. For example, in many contexts, mothers of malnourished children were identified as key intra-community resources as they were in the best position to demonstrate their struggle and success in relation to malnutrition and thus act as motivators of other women in a similar situation. Their engagement in communication strategies proved more efficient as the information was shared by community peers, making it automatically more believable and relevant for other community members.

### Analyze: Participatory Design

Community engagement efforts should not stop with data collection. CMN experience has shown that the community provides invaluable insight into the factors that encourage or dissuade them from attending services. Their insight often differs from that of program implementers, which means that service providers could act on an inaccurate interpretation of community behavior, unless further community engagement efforts are undertaken. For example, in the Democratic Republic of Congo, the heavy workload of women was ranked as a top barrier to access to CMAM services by community members, while it was positioned in the ninth place by the assessment team. This striking difference of perception demonstrates that any efforts designed to remove a key barrier identified by the assessment team would have been inaccurate and would fail to address the community’s prime concern.

For this reason, it is not only important to engage communities during the data analysis stage but also ensure their active participation during the solution design. To achieve both ends, the CMN advised to organize formal multi-stakeholder meetings, marked by the presence of local health system representatives and the most influential community figures, or less formal community debriefings at the health center level. The community members included, but were not limited to, traditional healers or birth attendants, community leaders, and representatives from women’s associations.

The more formal debriefings solicited feedback on the assessment findings from the community followed by a structured discussion around root causes and solutions for improving the program. The informal discussions are comprised of a community weighting exercise using flashcards, each with a booster or barrier depicted, so that the community could provide feedback on the perceived importance of the boosters and barriers. Both exercises provided an opportunity for the community and stakeholders to shape the analysis but also to take a lead role in identifying appropriate community-implemented solutions to overcome these barriers.

A concrete example of this comes from experiences in Mali, where NGOs conducted a series of formal multi-stakeholder consultations with the findings of SQUEAC assessments, which resulted in the development of context-specific recommendations. The community was pleased to be involved in discussing solutions to the issues identified, and importantly, this practice has set the tone for further interactions and collaboration among various actors. Moreover, the consultations were deemed a positive and informative experience for service delivery staff. So much so that at a recent national-level stakeholder workshop, service providers committed to sharing results of SQUEAC investigations with the communities. The aim of this interaction would be initiate a participatory collaboration process for the implementation of new activities or to further adjust them to the local context.

### Act: Participatory Implementation

In standard health service provision, health staff – nurses and community mobilizers – are recognized as “implementers” while the community as passive recipients of assistance. However, the CMAM model and the wider concept of community mobilization aim to encourage the community to take ownership of public health issues, which concern them and consequently of the programs put in place to address them. Considering their involvement in the problem and solution identification phase, they are likely well placed to implement certain activities and carry out their part of the program’s successes and failures – all this at a low cost, greater efficiency, and impact (14). After all, certain persistent barriers, such as seasonal barriers, poor awareness of malnutrition, or shortcomings in community screening, cannot be solved in an effective and sustainable manner without the community’s participation.

#### Seasonal Barriers Limiting Physical Access to Health Centers

In Mali, one SQUEAC investigation identified a low uptake of services and high defaulting in villages located far from the health facility, prone to additional seasonal barriers, such as flooded roads or high workload during the harvest season. Presented with these findings and sensitized about common health consequences of non-treatment of malnutrition or discontinuation of treatment, villagers mobilized themselves to establish a fund to finance the fuel for motorcycles, which then brought children to a health facility for free, especially during the rainy season, when difficulties of access are increased. By highlighting this to the community as a barrier to treatment and creating a space in which a solution could be identified, they were empowered to identify a way to overcome it.

#### Poor Awareness of Malnutrition

A SQUEAC investigation in Mali identified imams as a key communicator and influencer in the community. As a result, the community decided to reach out to them, resulting in a commitment to preaching on health and nutrition after prayers and to encourage men to bring their children to health facilities. A similar approach also proved successful in Chad, where imams and traditional healers played a crucial role in communication with communities and treatment acceptance. Following their interventions, male carers were more open to accept referrals and grant permission to their spouses to attend the program, which resolved a barrier previously impeding the uptake of services.

#### Shortcomings in Community Screening

As screening strategies are often based on activities of community volunteers, which frequently remain insufficient with regard to geographical and activity coverage, a program in Niger confided in carers of malnourished children to complement their efforts (15). The engagement of female volunteers with previous program experience added a new dimension to an interaction with the program and very quickly yielded results. Not only were women more invested but they also benefited from casual interactions with each other, during which they could screen children and refer them to health facilities. In addition, this activity was found to result in less refusals and more admissions of referred cases by the health center while equally empowering women to play a key role in improving their family’s and community’s health outcomes. Consequently, the community’s perception of the program and its eligibility criteria rose proportionately at minimal financial cost to the implementers.

## Outcomes

There are multiple benefits of this participatory approach to program management: it encourages implementers to gain a deeper understanding of social and cultural specificities of the context of their interventions while allowing the community to better understand the program or its “raison d’etre.” Alongside this knowledge, they have an opportunity to voice their perceptions of its strengths and weaknesses and to contribute to the development and implementations of solutions to address these weaknesses. Additionally, the approach is based on collective responsibility rather than placing the burden on individuals. With this comes the sharing of responsibility for the program’s successes and failures with implementers. In this respect, the assessment is only the beginning of the program’s engagement with served communities, allowing them to be part of the learning process and to secure their active participation in the service delivery. This rights-based approach to monitoring and evaluation drives both implementers (NGO and Ministry of Health) and the community to become equal partners, with the community gaining both rights and responsibilities rather than being passive beneficiaries (16). The low financial cost of these activities makes it a very accessible approach to program improvement, especially in resource-limited environments.

In reinforcing these methods to CMAM implementers, the CMN observed that both Ministry of Health and NGO staff have adjusted their understanding and approach to service provision. This is not only in recognition of the importance of integrating communities and those with specific needs but also in acknowledgment of the added value of working with communities, such as sharing the workload and responsibility. Concurrently, communities have proven their willingness and interest in being actively involved in facilitating program-strengthening activities, recognizing the public health implications of SAM. This has resulted in a stronger dialog, open information sharing channels between all stakeholders and local solutions to barriers that NGOs or Ministry of Health implementers were not in a position to tackle.

## Conclusion

This shift of focus is bringing the model back to its origin, placing the community at the center of implementation. Such a community-centered approach is critical if we are going to make sustained increases in the coverage of treatment services and reduce the burden. These experiences show that there is an appetite from implementers and communities in multiple contexts for a more participatory and collaborative approach to programing in order to respond to barriers of access and uptake of services.

## Author Contributions

LB, ER, CM, and SW conducted the field work as members of the Coverage Monitoring Network and drafted and approved the manuscript.

## Conflict of Interest Statement

The authors declare that the research was conducted in the absence of any commercial or financial relationships that could be construed as a potential conflict of interest.

## Footnotes

^1^High opportunity costs describe the decision made by carers not to attend treatment services because the direct and indirect costs and the implications of attending (e.g., travel costs, loss of income, loss of agricultural labour, etc.) are perceived as being too high.

^2^Inter-programme interface problems describes a child with SAM that seeks treatment or is already enrolled in a related treatment service at a health facility (e.g., malaria treatment) but is not identified as being eligible for SAM treatment and is not, therefore, transferred or referred for SAM treatment.

^3^Previous rejection from the programme describes a child with SAM that has previously attended the programme for screening or had been referred to treatment by programme staff or from another programme but was subsequently found not to meet the programme’s admission criteria and thus did not receive treatment at that time. Carers are then less willing to follow-up future referrals fearing further rejection.

^4^Burkina Faso, Chad, Democratic Republic of Congo, Ethiopia, Kenya, Mali, Niger, Pakistan, and South Sudan.

^5^For the most up-to date guidance on this qualitative phase, please refer to the tools on the Coverage Monitoring Website (specifically “How to Conduct a Community Assessment”)

^6^In CMAM communication strategies, experience of “struggle and success” refers to stories of children falling ill with malnutrition (“struggle”) and gradually healing (“success”) while in the programme. These stories often have a huge communication potential as carers’ personal experiences address other carer’s concerns in the most personal and relevant way.

## Abbreviations

CMAM, community-based management of acute malnutrition; CMN, Coverage Monitoring Network; CTC, community-based therapeutic care; MAM, moderate acute malnutrition; NGO, non-governmental organization; SAM, severe acute malnutrition; SLEAC, simplified lot quality assurance sampling evaluation of access and coverage; SQUEAC, semi-quantitative evaluation of access and coverage.
